# Autologous Tumor Lysate-Pulsed Dendritic Cell Immunotherapy with Cytokine-Induced Killer Cells Improves Survival in Gastric and Colorectal Cancer Patients

**DOI:** 10.1371/journal.pone.0093886

**Published:** 2014-04-03

**Authors:** Daiqing Gao, Changyou Li, Xihe Xie, Peng Zhao, Xiaofang Wei, Weihong Sun, Hsin-Chen Liu, Aris T. Alexandrou, Jennifer Jones, Ronghua Zhao, Jian Jian Li

**Affiliations:** 1 Biotherapy Center, Qingdao Center Hospital, The Second Affiliated Hospital, Qingdao University Medical College, Qingdao, China; 2 Department of Radiation Oncology, NCI-Designated Comprehensive Cancer Center, University of California at Davis Sacramento, Sacramento, California, United States of America; 3 Department of Medicine, University of Saskatchewan, Saskatoon, Canada; Johns Hopkins Hospital, United States of America

## Abstract

Gastric and colorectal cancers (GC and CRC) have poor prognosis and are resistant to chemo- and/or radiotherapy. In the present study, the prophylactic effects of dendritic cell (DC) vaccination are evaluated on disease progression and clinical benefits in a group of 54 GC and CRC patients treated with DC immunotherapy combined with cytokine-induced killer (CIK) cells after surgery with or without chemo-radiotherapy. DCs were prepared from the mononuclear cells isolated from patients using IL-2/GM-CSF and loaded with tumor antigens; CIK cells were prepared by incubating peripheral blood lymphocytes with IL-2, IFN-γ, and CD3 antibodies. The DC/CIK therapy started 3 days after low-dose chemotherapy and was repeated 3–5 times in 2 weeks as one cycle with a total of 188.3±79.8×10^6^ DCs and 58.8±22.3×10^8^ CIK cells. Cytokine levels in patients' sera before and after treatments were measured and the follow-up was conducted for 98 months to determine disease-free survival (DFS) and overall survival (OS). The results demonstrate that all cytokines tested were elevated with significantly higher levels of IFN-γ and IL-12 in both GC and CRC cohorts of DC/CIK treated patients. By Cox regression analysis, DC/CIK therapy reduced the risk of post-operative disease progression (p<0.01) with an increased OS (<0.01). These results demonstrate that in addition to chemo- and/or radiotherapy, DC/CIK immunotherapy is a potential effective approach in the control of tumor growth for post-operative GC and CRC patients.

## Introduction

Gastric cancer (GC) and colorectal cancer (CRC) are major malignant diseases of alimentary tract. While GC is the most common cancer in the Asian-Pacific region, CRC is ranked as the fourth most common malignancy world-wide, with about 1.2 million new cases and 609,051 deaths annually [1]. Surgical resection with or without adjuvant chemo- and/or radiation therapy remains the key modality for GC and CRC, but unfortunately shows limited clinical benefits due to high rate of tumor metastasis. Although current adjuvant chemo-radiation therapy has been shown to extend patient survival in the presence of recurrent lesions [2], [3], severe side effects usually limit the efficacy of this anti-cancer modality [2]–[4]. To further improve the overall survival for GC and CRC patients, it is critical to explore novel approaches to control tumor metastasis with or without the use of traditional chemo-and/or radiotherapy.

The dendritic cells (DCs) play a crucial role in the induction of antigen-specific T-cell responses to provide active immunotherapy [5]–[7]. Clinical studies using specifically designed DC-targeted cancer cell vaccines demonstrated different clinical benefits. Patients with lymphoma [8], [9], metastatic melanoma [10], [11], colon cancer, and non-small cell lung cancer [12] showed that vaccination with tumor antigen-pulsed DCs, either isolated directly from blood or generated *ex vivo* from blood precursors, elicited antigen specific immune reaction and, in some cases, significant tumor responses. In fact, application of an active immunotherapy regimen, Sipuleucel-T (APC8015) used by activating peripheral blood mononuclear cells (PBMCs) with a prostatic acid phosphatase (PAP), a fusion protein of prostate cancer antigen, with GM-CSF, resulted in approximately 4 month-prolonged median survival in prostate cancer patients [13]–[15], and was approved by FDA for the treatment of metastatic prostate cancers [14], [16], [17]. CIK cells are a subset of natural killer T lymphocytes (NKT) that are predominantly CD3^+^CD56^+^ type II NKT cells [18], and such cells can be generated *ex vivo* by incubating peripheral blood lymphocytes with an agonistic anti-CD3 monoclonal antibody, interleukin (IL)-2, IL1-β and interferon (IFN)-γ. CIK cells, supported by encouraging clinical trial results in both autologous and allogeneic contexts, are known to cytolytically eliminate tumor cells [19]. In contrast to lymphokine-activated killer (LAK) cells, which are cytotoxic effector T-cells stimulated predominantly in response to high concentration of interleukin-2 (IL-2), CIK cells exhibit enhanced tumor cell lytic activity [20], [21], higher proliferation rate [22], and relatively lower toxicity [23]. Although passive immunotherapy by adoptive transfer of T cells is believed to be effective in the control of primary tumors, it is unclear whether passive immunotherapy is effective in the long-term control of tumor relapse [24]. On the other hand, the active immunotherapy using tumor-specific vaccines, such as DC vaccine, has the potential benefit to significantly enhance tumor-specific effector and memory T cells. The anti-tumor responses triggered by DC/CIK therapy have been reported in a number of *ex vivo* [25]–[29] and *in vivo* [30] studies as well as in preliminary clinical trials in patients with non-Hodgkin's and Hodgkin's lymphoma [31], [32] and non-small cell lung cancer with few side effects [33]. In the present study, clinical benefits are evaluated in a group of 54 GC and CRC patients treated with DC immunotherapy combined with cytokine-induced killer (CIK) cells after surgery with or without chemo-radiotherapy. The results demonstrate improved rates of DFS and OS with elevated levels of IFN-γ and IL-12 in both GC and CRC cohorts of DC/CIK treated patients.

## Patients and Methods

### Study design, patient recruitment, and data collection

We conducted the study with patients treated in the Department of Surgery and Center of Biological Therapy, Qingdao Central Hospital, Qingdao, China from 2005 to 2010 (Table 1). Patients were recruited with the following criteria: 1) 18 years and older, 2) pathologically confirmed GC or CRC, 3) underwent surgical resection of primary tumors, 4) no evidence of tumor metastasis or recurrence before receiving cell-based immunotherapy, 5) having completed chemo-and/or radiotherapy for at least 1 month, and 6) signing the consent form. Patients in the control group were recruited with demographic and clinical pathological characteristics. A total of 27 patients were randomly assigned to cell-based immunotherapy and 27 patients were included in the control group. Tumors were staged according to the International Union Against Cancer's (UICC) classification based on pTNM subsets. All patients' informed consents were obtained and the procedures of the study were reviewed and approved by the Ethic Review Board of Qingdao Central Hospital. After cell-based immunotherapy, all patients obtained follow-up by hospital visits and/or telephone interviews in at least every 6 months. Local tumor recurrence and distant metastasis were examined by imaging analyses. DFS (disease-free survival, the time interval between surgery and tumor recurrence) and OS (overall survival, the time between surgery and last follow-up) were collected in the project research database. DFS and OS rates were calculated in both treatment and control groups from the dates of surgery and the follow-ups were completed for all patients by August 2011, including the patients with 12 month or longer period after immunotherapy.

**Table 1:** Demographic and pathological data of patients recruited for treatment and control groups*

	Treatment	Control	P
Age	61.56±12.82	64.48±12.77	0.41
Sex (male/female)	16/11	16/11	1.00
Diagnosis (gastric/colorectal)	14/13	14/13	1.00
Differentiation (well/moderate/poor)	13/14	14/13	0.76
Tumor (T1-2/T3-4)	6/21	4/23	0.73
Lymph nodes (N negative/N positive)	7/20	15/12	0.03
TNM stage (I II/III IV)	5/22	16/11	<0.01
Radiotherapy	0	0	1.00
Chemotherapy	13	22	0.04
Radiotherapy and Chemotherapy	1	5	0.02
Metastasis/recurrence(gastric/colorectal)	2/5	7/12	<0.05

*All patients were recruited as control and treatment groups following the protocol approved by Ethics Committee at Qingdao Central Hospital Informed Consent).

### DC vaccine and CIK cellular therapy

A dosage of 1×10^6^ units IL-2 (Quangang Pharma Co. Shandong, China) in 250 ml physiological saline was prepared and intravenously administrated to patients in the treatment group, once a day for 5 consecutive days. Three to six days post-IL-2 injection, mononuclear cells (4–6×10^9^) were collected from total 6–9 liters of circulating blood by COBE Spectra Apheresis System (Gambro BCT, Inc., Colorado, USA) and stored in 120–150 ml plasma. If the mononuclear cells were less than 4×10^8^/Liter, GM-CSF (150 μg) were subcutaneously applied to the patients—once a day for 1–3 days before further collection of mononuclear cells was processed.

### Antigen preparation

Tumor antigen was prepared by following the established protocol [34], [35] from human AGS gastric cancer or LS-174T colon cancer cells. Tumor cells were cultured for 2–3 passages (1–2×10^8^), and collected, and washed with normal saline for 3 times, and lysed by freezing-thawing three times, and analyzed with ultrasonic cell disruption. Lysates from tumor cells were then fractionated by centrifugation (1200 rpm × 5 min), and the supernatant was collected and filtered with a 0.22 filter (Carrighwohill, Co. Cork, Ireland), and protein concentration in the supernatant was measured before storage at −80°C.

### Preparation of DC and CIK cells

Mononuclear cells were isolated from the collected cells from peripheral blood of GC and CRC patient by following an established method [36], [37] using Ficoll (GE Healthcare life Science, Shanghai, China). Isolated cells were suspended in RPMI 1640 medium at a concentration of 1×10^7^ mL^−1^ and cultured in 175 cm^2^ cell culture flasks at 37°C, 5% CO_2_ for 2 hrs. Adherent cells were cultured in serum free DC culture medium (CellGenix, Freiburg, Germany) containing 20 ng/mL interleukin 4 (IL-4) and 50 ng/mL GM-CSF at 37°C, 5% CO_2_ for 6 days. DC culture medium containing prepared tumor antigen was then added in cultured cells to a final concentration of 50 μg/ml, and cultured for another 2 days. Matured DCs were examined by flow cytometry for DC markers CD83 and HLA-DR. Preparations were tested as bacteria and pyrogen free. A portion of the prepared DC was infused into the patients and the remaining DCs were stored at −80°C. Suspension cells were cultured in RPMI 1640 medium (2×10^6^/mL) containing 10% BFS, 100 U/mL IL-2, 300 U/mL IFN-γ, 20 ng/mL CD3 monoclonal antibody (R&D Systems, Minneapolis, MN, USA), and 40 U/mL gentamycin. On day 8, a portion of the cells were examined by flow cytometry for CIK cell markers with antibodies against CD3, CD8, and CD56. Cells were then kept in normal saline with 1% albumin, and the rest of suspensions cells were kept in culture for another 7 days.

### DC vaccine and CIK cell administration

**Figure 1:**
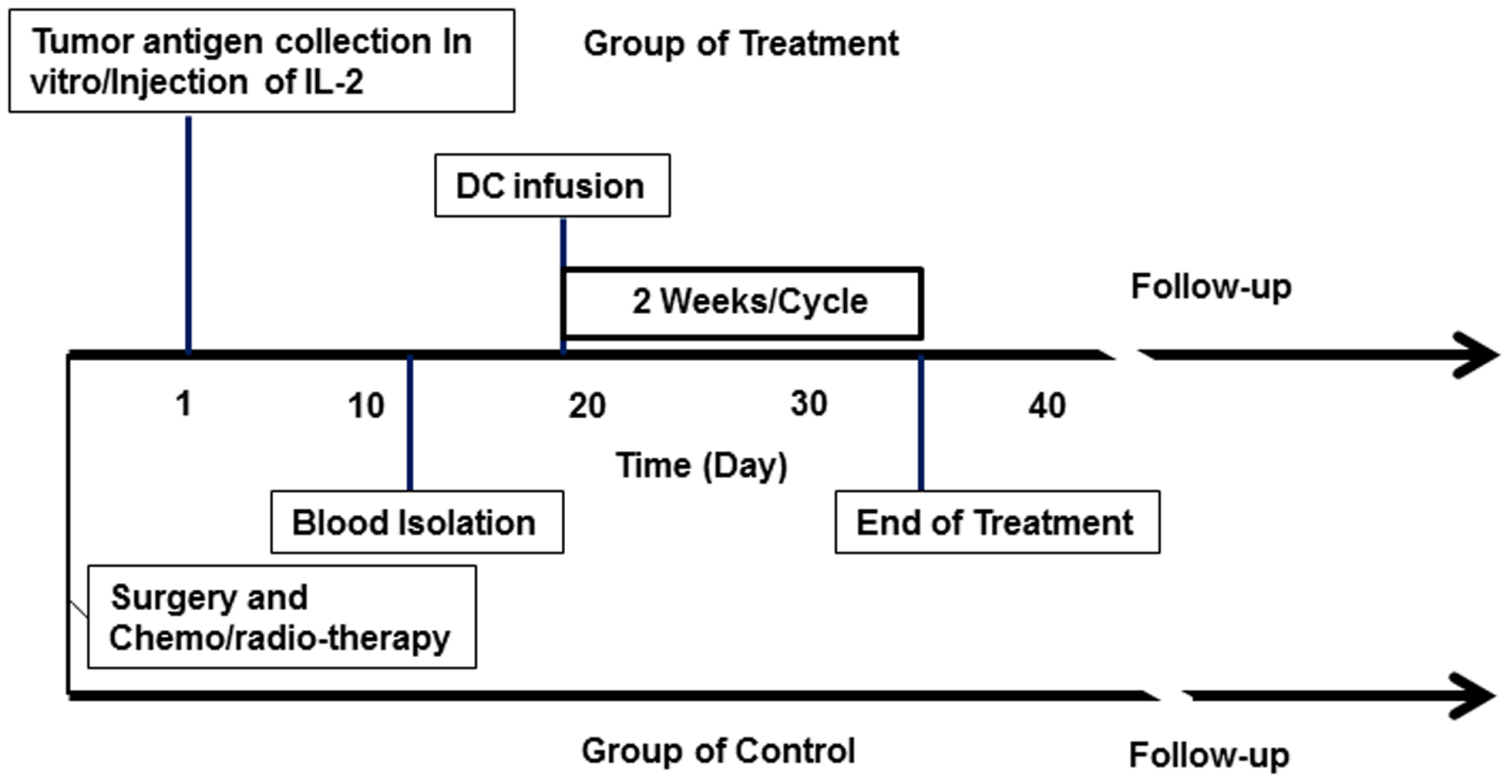
Following up of DC/CIK therapy and control patients. The prognoses of patients were recorded up to 98 months after the dates of surgery in both treatment and control groups.

Patients in the treatment group received one cycle of low dose chemotherapy starting on the day after mononuclear cells collection with Carmofure (100 mg, po., bid) for 5∼6 days. The infusion of DCs and CIK cells was started on day 2 or day 3 after chemotherapy. Prepared DC cells were divided into two parts: one part was mixed with CIK cells in 250 ml normal saline containing 1500 U/mL IL-2 and 1% albumin, and infused into the patients intravenously. The other part was suspended in 1.5 ml normal saline and injected subcutaneously into the area of draining lymph nodes adjacent to the tumor sites. The treatment was repeated 3–5 times in 2 weeks as one cycle. Patients with advanced stage diseases received 2 cycles of the treatment, while early stage patients were treated with one cycle. The immune responses of patients were monitored before and after the treatment by measuring serum levels of IFN-γ, IL-2, IL-6, IL-10, and IL-12 determined by ELISA (R&D systems, Minneapolis, MN, USA). The time schedules of DC and CIK preparation and infusion were shown in Figure 1.

### Statistical analysis

Data was presented as percentages, means with standard deviation (mean ± SD), or median with 95% confidence intervals (95% CI). Chi square test, Student's t-test or Mann–Whitney test, Pearson's linear or Spearman rank tests were used for analysis as appropriate. Treatment outcome was analyzed by Kaplan–Meier survival curves with log rank test, and multivariate Cox proportional hazards regression tests. Statistical significance was set at p<0.05. All statistical analyses were performed using SPSS 16.0 for Windows (SPSS, Chicago, IL, USA).

## Results

### Baseline information

A total of 54 patients with histological confirmed gastric or colorectal adenocarcinoma were randomly recruited into this study consisting of 27 patients in each group. The demographic and clinical pathological data of patients in the treatment and control groups were presented in Table 1. Half of the patients in the treatment group and all patients in control group had received at least one cycle of chemo-radiation before enrollment. Immunotherapy was initiated at least one month after the termination of chemo- and/or radiation therapy and follow-up time was 98 months for all patients in treatment and control groups.

### Characteristics of DC/CIK cells for cell infusion

**Figure 2:**
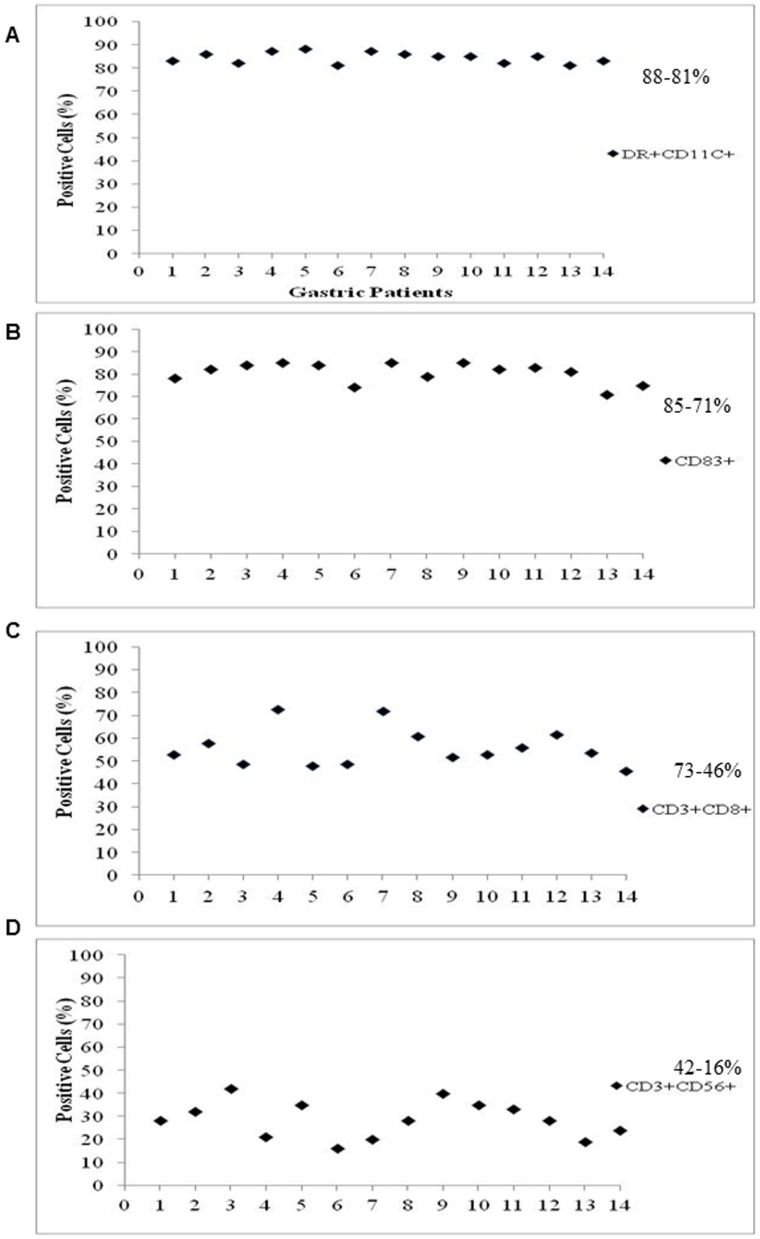
The maturity of dendritic cells (A,C) and CIK cells (B, D) was determined by measuring the percentage of cells expressing DR and CD11c, CD83 (DC markers), and CD3/CD8, CD3/CD56 (CIK cell markers) in blood samples collected from treated gastric cancer patients.

**Figure 3:**
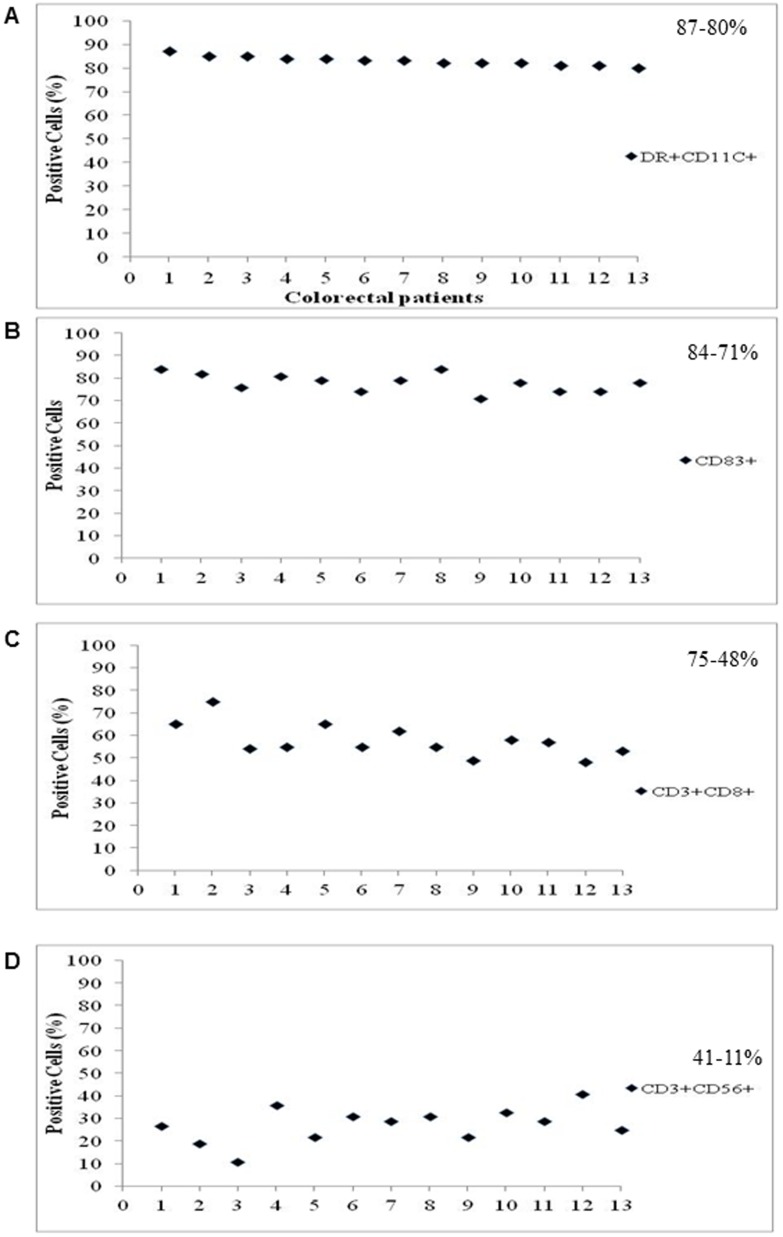
The maturity of dendritic cells (A,C) and CIK cells (B, D) was determined by measuring the percentage of cells expressing DR and CD11c, CD83 (DC markers), and CD3/CD8, CD3/CD56 (CIK cell markers) in blood samples collected from treated colorectal cancer patients.

The average number of cells infused into patients in one cycle was 188.3±79×10^6^ for DCs and 58.8±22.3×10^8^ for CIK cells. Cells expressing DR/CD11C and CD83 (DC markers) or CD3/CD8 and CD3/CD56 (CIK markers) were analyzed. DCs prepared from GC patients were 88%-81% positive for DR/CD11C and 85%-71% positive for CD83 (Fig. 2A, B). CIK cells prepared from GC patients showed 73%-46% positive for CD3/CD8 and 42%-16% positive for CD3/CD56 (Fig. 2C, D). In addition, DC prepared from CRC patients were 87%-80% positive for DR/CD11C and 84%-71% positive for CD83 (Fig. 3A, B), and CIK cells from CRC patients were 75%-48% positive for CD3/CD8 and 41%-11% positive for CD3/CD56 respectively (Fig. 3C, D). The overall cytolytic efficacy of CIK cells from all patients was enhanced ∼2.5 fold. These results indicate that the majority of the DCs and CIK cells prepared from patients were mature and functional.

### Serum cytokine levels after DC/CIK therapy

**Figure 4:**
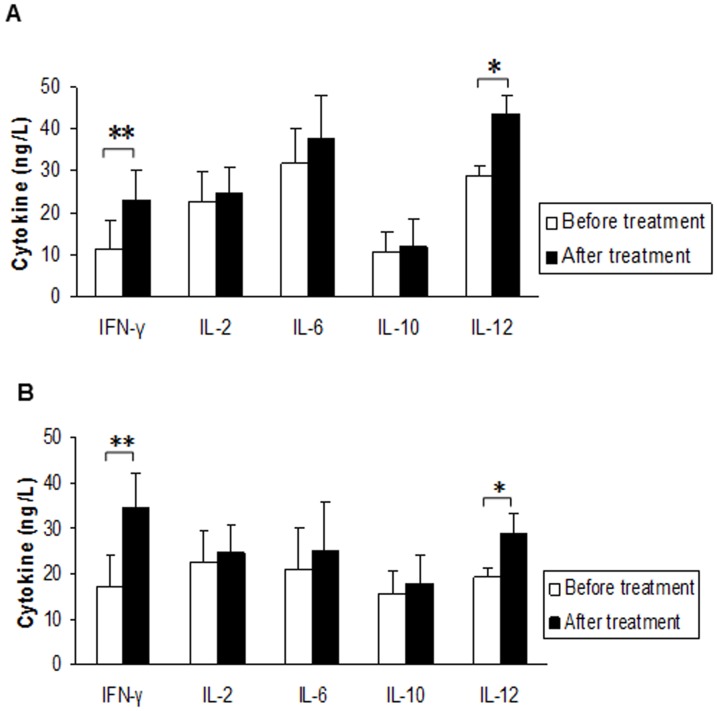
Serum levels of INF-γ, IL-2, IL-6, IL-10 and IL-12 measured from gastric (A) and colorectal (B) cancer patients before and after a 2-week cycle of treatment with DC/CIK cells (A, n = 14, B, n = 13, *p<0.05, **p<0.01).

We examined serum levels of a group of cytokine for each patient before and after DC/CIK treatments (Figure 4 for GC (A) and CRC (B)). The levels of IL-2, IL-6 and IL-10 slightly enhanced, with no statistical significance in the sera of GC and CRC patients (Fig. 4A, B). However, serum IL-12 and IFN-γ levels were significantly increased in both GC and CRC patients, indicating that DC/CIK therapy may specifically promote a Th1 immune response to mediate tumor killing effect of DC/CIK therapy.

### Disease-free survival (DFS) and overall survival (OS) of patients after cell-based immunotherapy

**Figure 5:**
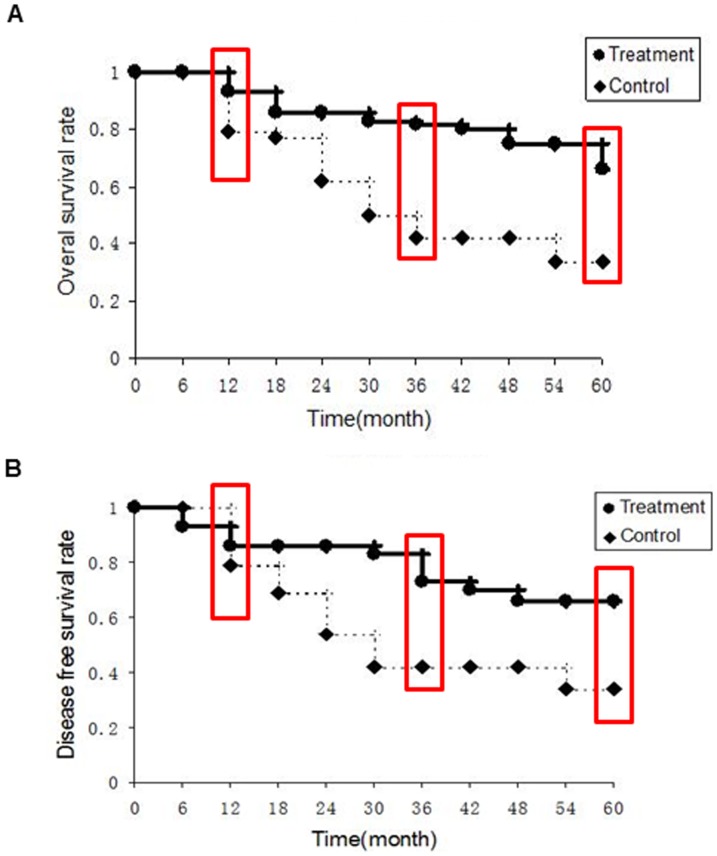
(A) The overall survival rate of the gastric cancer patients. The 1-, 3-, and 5-year survival rates in the treatment group was 93%, 82%, and 66%, compared with the control 79%, 42%, and 34% respectively. B) Disease free survival rate of the gastric cancer patients. The 1-, 3-, and 5-year survival rates in the treatment groups (red box marked) were 86%, 73%, and 66%, compared with the control 79%, 42%, and 34% respectively. The cumulative survival curves in A and B were analyzed by the Kaplan-Meier method.

**Figure 6:**
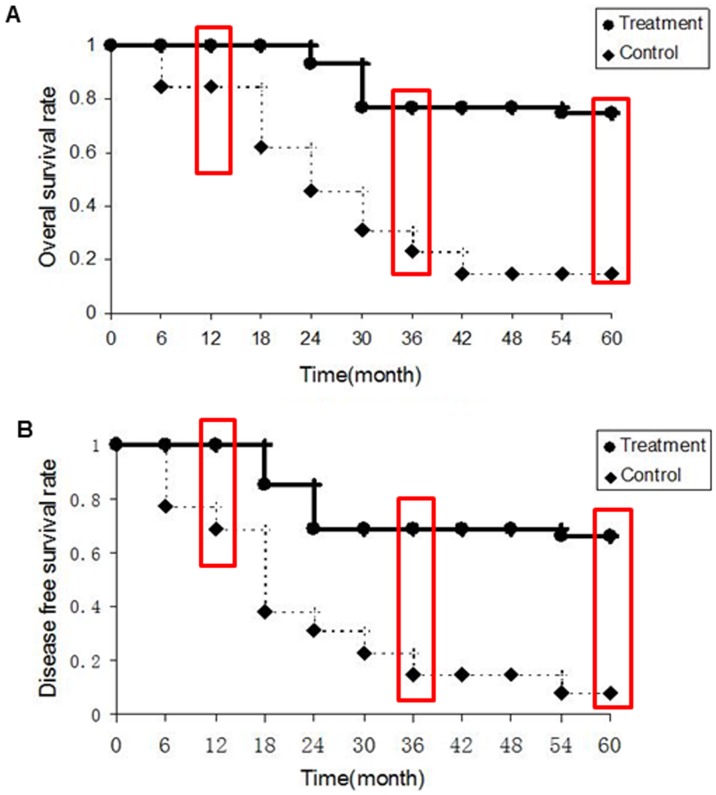
(A) The overall survival rate of the colorectal cancer patients. The 1-, 3-, and 5-year survival rates in the treatment group were 100%, 77%, and 75%, compared with the control 85%, 23%, and 15% respectively. (B) Disease free survival of the gastric cancer patients. The 1-, 3-, and 5-year survival rates in the treatment group were 100%, 69%, and 66%, compared with the control 69%, 15%, and 8% respectively. The cumulative survival curves in A and B were analyzed by the Kaplan-Meier method.

As shown in Figures 5 and 6, DFS and OS were both significantly prolonged in patients in DC/CIK treatment groups (GC: 5-year DFS rate: 66%, OS rate: 66%; CRC: 5-year DFS rate: 66%, OS rate: 75%) compared with the patients in control groups (GC: 5-year DFS rate: 34%, OS rate: 34%; CRC: 5-year DFS rate: 8%, OS rate: 15%;p<0.01). To examine the differential response of GC and CRC, we analyzed two cohorts (GC: p<0.05; CRC: p<0.01) and found that CRC are more sensitive to DC/CIK therapy than GC. In addition, analysis with multivariate Cox proportional regression confirmed that DC/CIK therapy significantly and independently reduced the risk of post-operative disease progression (Odd ratio: 0.09, 95% CI: 0.02–0.42; p<0.01) or patient deceases (Odd ratio: 0.05, 95% CI: 0.01–0.37; p<0.01) after adjusting for age, sex, tumor grade, TNM stages, and previous chemo- and/or radiation treatments.

### Potential adverse effects of cell-based immunotherapy

The most common adverse effect observed in all patients receiving DC/CIK therapy was fever, which occurred in 9 of 27 treated patients (33%) in the range of 37.5–40°C. All patients recovered spontaneously or after antipyretic treatment with non-steroid medicine. No other significant complications accompanying cell-based immunotherapy were observed.

## Discussion

Cancer immunotherapy had shown a potential efficacy in tumor growth control and patient survival [14], [38], [39] as the news released that “Instead of using surgery, chemotherapy, or radiotherapy, researchers from the National Institutes of Health are finding so-far limited but inspiring success in a new approach for fighting cancer, using the immune system to attack the tumors the way it would be a cold or flu. -CNN.com (August 2006)”. Although extensively studied in cells and animal models, the clinical data regarding the exact benefit of immunotherapy in patient survival and disease progression remain to be further investigated [17], [40]. Despite limited size of cohorts, this study demonstrates a remarkable enhancement in the post-surgical control of tumor recurrence and survival rates in GC and CRC patients treated with combined DC/CIK therapy. It has been well established that the DCs prime naïve T-cells and DC vaccine combined with CIK cell therapy has achieved encouraging promise as a novel therapeutic approach for disease control in specific cancers [41]. DCs [6], [7], [42] have the capability to present tumor antigens to T lymphocytes and induce the specific cytotoxic T cells against tumor antigens [43]. Sipuleucel-T, the first DC vaccine, was approved for clinical application by FDA in USA to treat asymptomatic metastatic castrate-resistant prostate cancer, improving patients' OS in phase III trial [14]. Additional promising results were reported in recent phase III trials using tumor vaccine to treat various late stage cancers [44], including melanoma, follicular lymphoma, CRC, and NSCLC. Studies in the clinics have established that DCs capture and process tumor-associated antigens and secrete cytokines to initiate an immune response [45].

In addition to DC vaccine, CIKs are induced by cytokines and possess nonspecific cytotoxicity against tumors [24]. CIK cells can kill tumor cells directly, but have significantly short term anti-cancer efficacy and they are less likely to control tumor outgrowth in the long-term [24]. In contrast, DC vaccine is shown to induce tumor-specific effector and memory T cells [24]. Therefore, the combination of DC vaccine with CIK treatment may have a potential higher cytotoxic activity and specificity in the effector T cells, which shows both short and long term anti-tumor efficacy. In agreement with the clinical benefits observed in other clinical trials[44], our study suggests that DC/CIK treatment can significantly enhance patient survival, prompting its future clinical investigation.

Although a number of pro-inflammatory cytokines were elevated in the DC/CIK treated patients, only two Th1 cytokines, IL-12 and IFN-γ, showed a significant increase in patients' sera in our study. Studies demonstrated that IFN-γ and IL-12 play critical roles in immunotherapy using DC and CIK [28], [33]. Tumor cells are highly heterogeneous [46] and a specific tumor may contain cells with both high and low MHC-I populations [47], [48]. Interestingly, MHC-I expression shows heterogeneous among tumor cells and radiation promotes the immunological recognition of the tumor cells by immune cells via MHC-I [49]. However, probably due to the heterogeneity in a given tumor, a single type of immune therapy may only be effective in a subpopulation of cancer patients. Similarly, tumor cells with higher MHC-I expression may be more sensitive to DC vaccine therapy while cells with lower MHC-I expression may be killed by CIK. Our results support that combined DC/CIK therapy may promote tumor cell cytotoxicity by targeting different populations of tumor cells, such as those with various levels of MHC-I.

The doses of DC and CIK to the patients in this study were determined based on previous dose escalating test, and found to be safe in all patients. It should be noted that in the current study, data were derived from patients treated with a modified regimen, i.e., before lymphocyte collection by intravenously administration of IL-2 to improve function and number of lymphocytes, which may contribute a significant role in DC/CIK therapy. In addition, patients also received 1 cycle of low dose chemotherapy prior to the DC and CIK infusion, which may also affect the overall effectiveness of DC/CIK therapy. We hypothesized that a 3-day gap is critical for sufficient “washout” of chemotherapeutic agent, so as not to affect the effectiveness of infused DC and CIK. Patients in the control group had been previously treated with at least 1 cycle of chemotherapy, comparable to patients enrolled in the immunotherapy group (Table 1 and Figures 5, 6). The potential effects of low dose chemotherapy before cell infusion [28] may also induce tumor cell killing, up-regulate the expression of tumor antigen and contribute to the composition of host immune cells. Therefore, further studies will be conducted with larger patient cohorts. In conclusion, this clinical study indicates that the combination of DC vaccine and CIK therapy may significantly improve the disease-free survival in gastric and colorectal cancer patients.

## References

[1] Jemal A, Bray F, Center MM, Ferlay J, Ward E, et al (2011) Global cancer statistics. CA Cancer J Clin 61: 69–90. 21296855 10.3322/caac.20107

[2] Ottoman RE, Langdon EA, Rochlin DB, Smart CR (1963) Side-Effects of Combined Radiation and Chemotherapy in the Treatment of Malignant Tumors. Radiology 81: 1014–1017. 14101708 10.1148/81.6.1014

[3] Palesty JA, Wang W, Javle MM, Yang GY (2004) Side effects of therapy: case 3. Gastric cancer after radiotherapy of pediatric Hodgkin's disease. J Clin Oncol 22: 2507–2509. 15197215 10.1200/JCO.2004.09.168

[4] Boulikas T, Vougiouka M (2004) Recent clinical trials using cisplatin, carboplatin and their combination chemotherapy drugs (review). Oncol Rep 11: 559–595. 14767508

[5] Frankenberger B, Schendel DJ (2012) Third generation dendritic cell vaccines for tumor immunotherapy. Eur J Cell Biol 91: 53–58. 21439674 10.1016/j.ejcb.2011.01.012

[6] Steinman RM, Banchereau J (2007) Taking dendritic cells into medicine. Nature 449: 419–426. 17898760 10.1038/nature06175

[7] Melief CJ (2007) Cancer: immune pact with the enemy. Nature 450: 803–804. 18026088 10.1038/nature06363

[8] Hsu FJ, Benike C, Fagnoni F, Liles TM, Czerwinski D, et al (1996) Vaccination of patients with B-cell lymphoma using autologous antigen-pulsed dendritic cells. Nat Med 2: 52–58. 8564842 10.1038/nm0196-52

[9] Timmerman JM, Czerwinski DK, Davis TA, Hsu FJ, Benike C, et al (2002) Idiotype-pulsed dendritic cell vaccination for B-cell lymphoma: clinical and immune responses in 35 patients. Blood 99: 1517–1526. 11861263 10.1182/blood.v99.5.1517

[10] Nestle FO, Alijagic S, Gilliet M, Sun Y, Grabbe S, et al (1998) Vaccination of melanoma patients with peptide- or tumor lysate-pulsed dendritic cells. Nat Med 4: 328–332. 9500607 10.1038/nm0398-328

[11] Thurner B, Haendle I, Roder C, Dieckmann D, Keikavoussi P, et al (1999) Vaccination with mage-3A1 peptide-pulsed mature, monocyte-derived dendritic cells expands specific cytotoxic T cells and induces regression of some metastases in advanced stage IV melanoma. J Exp Med 190: 1669–1678. 10587357 10.1084/jem.190.11.1669 PMC2195739

[12] Fong L, Hou Y, Rivas A, Benike C, Yuen A, et al (2001) Altered peptide ligand vaccination with Flt3 ligand expanded dendritic cells for tumor immunotherapy. Proc Natl Acad Sci U S A 98: 8809–8814. 11427731 10.1073/pnas.141226398 PMC37517

[13] Higano CS, Schellhammer PF, Small EJ, Burch PA, Nemunaitis J, et al (2009) Integrated data from 2 randomized, double-blind, placebo-controlled, phase 3 trials of active cellular immunotherapy with sipuleucel-T in advanced prostate cancer. Cancer 115: 3670–3679. 19536890 10.1002/cncr.24429

[14] Kantoff PW, Higano CS, Shore ND, Berger ER, Small EJ, et al (2010) Sipuleucel-T immunotherapy for castration-resistant prostate cancer. N Engl J Med 363: 411–422. 20818862 10.1056/NEJMoa1001294

[15] Higano CS, Small EJ, Schellhammer P, Yasothan U, Gubernick S, et al (2010) Sipuleucel-T. Nat Rev Drug Discov 9: 513–514. 20592741 10.1038/nrd3220

[16] Huber ML, Haynes L, Parker C, Iversen P (2012) Interdisciplinary critique of sipuleucel-T as immunotherapy in castration-resistant prostate cancer. J Natl Cancer Inst 104: 273–279. 22232132 10.1093/jnci/djr514 PMC3283534

[17] Frohlich MW (2012) Sipuleucel-T for the treatment of advanced prostate cancer. Semin Oncol 39: 245–252. 22595047 10.1053/j.seminoncol.2012.02.004

[18] Gutgemann S, Frank S, Strehl J, Schmidt-Wolf IG (2007) Cytokine-induced killer cells are type II natural killer T cells. Ger Med Sci 5: Doc07. 19675715 PMC2703238

[19] Mesiano G, Todorovic M, Gammaitoni L, Leuci V, Giraudo Diego L, et al (2012) Cytokine-induced killer (CIK) cells as feasible and effective adoptive immunotherapy for the treatment of solid tumors. Expert Opin Biol Ther 12: 673–684. 22500889 10.1517/14712598.2012.675323

[20] Lu PH, Negrin RS (1994) A novel population of expanded human CD3+CD56+ cells derived from T cells with potent in vivo antitumor activity in mice with severe combined immunodeficiency. J Immunol 153: 1687–1696. 7519209

[21] Margolin KA, Negrin RS, Wong KK, Chatterjee S, Wright C, et al (1997) Cellular immunotherapy and autologous transplantation for hematologic malignancy. Immunol Rev 157: 231–240. 9255634 10.1111/j.1600-065x.1997.tb00986.x

[22] Linn YC, Hui KM (2003) Cytokine-induced killer cells: NK-like T cells with cytotolytic specificity against leukemia. Leuk Lymphoma 44: 1457–1462. 14565644 10.3109/10428190309178764

[23] Hontscha C, Borck Y, Zhou H, Messmer D, Schmidt-Wolf IG (2011) Clinical trials on CIK cells: first report of the international registry on CIK cells (IRCC). J Cancer Res Clin Oncol 137: 305–310. 20407789 10.1007/s00432-010-0887-7 PMC11828187

[24] Thanendrarajan S, Nowak M, Abken H, Schmidt-Wolf IG (2011) Combining cytokine-induced killer cells with vaccination in cancer immunotherapy: more than one plus one? Leuk Res 35: 1136–1142. 21652069 10.1016/j.leukres.2011.05.005

[25] Gonzalez-Carmona MA, Marten A, Hoffmann P, Schneider C, Sievers E, et al (2006) Patient-derived dendritic cells transduced with an a-fetoprotein-encoding adenovirus and co-cultured with autologous cytokine-induced lymphocytes induce a specific and strong immune response against hepatocellular carcinoma cells. Liver Int 26: 369–379. 16584401 10.1111/j.1478-3231.2005.01235.x

[26] Wongkajornsilp A, Sangsuriyong S, Hongeng S, Waikakul S, Asavamongkolkul A, et al (2005) Effective osteosarcoma cytolysis using cytokine-induced killer cells pre-inoculated with tumor RNA-pulsed dendritic cells. J Orthop Res 23: 1460–1466. 15908161 10.1016/j.orthres.2005.03.009.1100230632

[27] Marten A, Greten T, Ziske C, Renoth S, Schottker B, et al (2002) Generation of activated and antigen-specific T cells with cytotoxic activity after co-culture with dendritic cells. Cancer Immunol Immunother 51: 25–32. 11845257 10.1007/s00262-001-0251-5 PMC11032866

[28] Marten A, Ziske C, Schottker B, Weineck S, Renoth S, et al (2001) Transfection of dendritic cells (DCs) with the CIITA gene: increase in immunostimulatory activity of DCs. Cancer Gene Ther 8: 211–219. 11332992 10.1038/sj.cgt.7700292

[29] Su X, Zhang L, Jin L, Ye J, Guan Z, et al (2010) Coculturing dendritic cells with zoledronate acid efficiently enhance the anti-tumor effects of cytokine-induced killer cells. J Clin Immunol 30: 766–774. 20549316 10.1007/s10875-010-9434-1

[30] Chan JK, Hamilton CA, Cheung MK, Karimi M, Baker J, et al (2006) Enhanced killing of primary ovarian cancer by retargeting autologous cytokine-induced killer cells with bispecific antibodies: a preclinical study. Clin Cancer Res 12: 1859–1867. 16551871 10.1158/1078-0432.CCR-05-2019

[31] Sun Y, Chen J, Cai P, Hu YH, Zhong GC, et al (2010) [Therapy of relapsed or refractory non-Hodgkin's lymphoma by antigen specific dendritic cells-activated lymphocytes]. Zhongguo Shi Yan Xue Ye Xue Za Zhi 18: 219–223. 20137151

[32] Leemhuis T, Wells S, Scheffold C, Edinger M, Negrin RS (2005) A phase I trial of autologous cytokine-induced killer cells for the treatment of relapsed Hodgkin disease and non-Hodgkin lymphoma. Biol Blood Marrow Transplant 11: 181–187. 15744236 10.1016/j.bbmt.2004.11.019

[33] Li H, Wang C, Yu J, Cao S, Wei F, et al (2009) Dendritic cell-activated cytokine-induced killer cells enhance the anti-tumor effect of chemotherapy on non-small cell lung cancer in patients after surgery. Cytotherapy 11: 1076–1083. 19929470 10.3109/14653240903121252

[34] Pan Y, Zhang J, Zhou L, Zuo J, Zeng Y (2006) In vitro anti-tumor immune response induced by dendritic cells transfected with EBV-LMP2 recombinant adenovirus. Biochem Biophys Res Commun 347: 551–557. 16842756 10.1016/j.bbrc.2006.05.214

[35] Pan Y, Chefalo P, Nagy N, Harding C, Guo Z (2005) Synthesis and immunological properties of N-modified GM3 antigens as therapeutic cancer vaccines. J Med Chem 48: 875–883. 15689172 10.1021/jm0494422 PMC3180873

[36] Moiseyenko V, Imyanitov E, Danilova A, Danilov A, Baldueva I (2007) Cell technologies in immunotherapy of cancer. Adv Exp Med Biol 601: 387–393. 17713028 10.1007/978-0-387-72005-0_42

[37] Schadendorf D, Algarra SM, Bastholt L, Cinat G, Dreno B, et al (2009) Immunotherapy of distant metastatic disease. Ann Oncol 20 Suppl 6vi41–50. 19617297 10.1093/annonc/mdp253 PMC2712591

[38] Morse MA, Deng Y, Coleman D, Hull S, Kitrell-Fisher E, et al (1999) A Phase I study of active immunotherapy with carcinoembryonic antigen peptide (CAP-1)-pulsed, autologous human cultured dendritic cells in patients with metastatic malignancies expressing carcinoembryonic antigen. Clin Cancer Res 5: 1331–1338. 10389916

[39] O'Neill D, Bhardwaj N (2005) Exploiting dendritic cells for active immunotherapy of cancer and chronic infection. Methods Mol Med 109: 1–18. 15585909

[40] Madan RA, Schwaab T, Gulley JL (2012) Strategies for optimizing the clinical impact of immunotherapeutic agents such as sipuleucel-T in prostate cancer. J Natl Compr Canc Netw 10: 1505–1512. 23221788 10.6004/jnccn.2012.0156 PMC6599514

[41] O'Neill DW, Bhardwaj N (2007) Exploiting dendritic cells for active immunotherapy of cancer and chronic infections. Mol Biotechnol 36: 131–141. 17914192 10.1007/s12033-007-0020-6

[42] Banchereau J, Steinman RM (1998) Dendritic cells and the control of immunity. Nature 392: 245–252. 9521319 10.1038/32588

[43] van Broekhoven CL, Parish CR, Demangel C, Britton WJ, Altin JG (2004) Targeting dendritic cells with antigen-containing liposomes: a highly effective procedure for induction of antitumor immunity and for tumor immunotherapy. Cancer Res 64: 4357–4365. 15205352 10.1158/0008-5472.CAN-04-0138

[44] Schlom J (2012) Recent advances in therapeutic cancer vaccines. Cancer Biother Radiopharm 27: 2–5. 22251077 10.1089/cbr.2012.1200 PMC3282014

[45] Marten A, Ziske C, Schottker B, Renoth S, Weineck S, et al (2001) Interactions between dendritic cells and cytokine-induced killer cells lead to an activation of both populations. J Immunother 24: 502–510. 11759073 10.1097/00002371-200111000-00007

[46] Duru N, Fan M, Candas D, Menaa C, Liu HC, et al (2012) HER2-associated radioresistance of breast cancer stem cells isolated from HER2-negative breast cancer cells. Clin Cancer Res 18: 6634–6647. 23091114 10.1158/1078-0432.CCR-12-1436 PMC3593096

[47] Li CD, Zhang WY, Li HL, Jiang XX, Zhang Y, et al (2005) Mesenchymal stem cells derived from human placenta suppress allogeneic umbilical cord blood lymphocyte proliferation. Cell Res 15: 539–547. 16045817 10.1038/sj.cr.7290323

[48] Beutler N, Hauka S, Niepel A, Kowalewski DJ, Uhlmann J, et al (2013) A natural tapasin isoform lacking exon 3 modifies peptide loading complex function. Eur J Immunol 43: 1459–1469. 23519916 10.1002/eji.201242725

[49] Sharma A, Bode B, Wenger RH, Lehmann K, Sartori AA, et al (2011) gamma-Radiation promotes immunological recognition of cancer cells through increased expression of cancer-testis antigens in vitro and in vivo. PLoS One 6: e28217. 22140550 10.1371/journal.pone.0028217 PMC3226680

